# Electrochemical Preparation and Post-treatment of Composite Porous Foam NiZn Alloy Electrodes with High Activity for Hydrogen Evolution

**DOI:** 10.1038/s41598-018-33205-4

**Published:** 2018-10-10

**Authors:** Jingguo Zhang, Youzhi Zhou, Shaoming Zhang, Shuo Li, Qiang Hu, Ligen Wang, Limin Wang, Fei Ma

**Affiliations:** 1 Beijing General Research Institute For Nonferrous Metals, Beijing, 100088 China; 2 GRIPM Advanced Materials Co.,Ltd, Beijing, 101407 China

## Abstract

Composite porous foam NiZn alloy electrodes with nano pore structure were prepared by the combination of eletrodeposition, heat treatment and HCl etching. The morphology of the electrodes was examined by scanning electron microscopy (SEM). And the component of the electrodes was analyzed by Energy Dispersive Spectrum (EDS). The specific surface area and pore size of the electrode were investigated by nitrogen adsorption. The phase constituents were analyzed by X ray diffraction (XRD), and the electrocatalytic characteristics for hydrogen evolution reaction of the electrodes in 30% (mass fraction) KOH solution were investigated by cathode polarization curve. The experimental results showed that the pores were formed on surface of the foam NiZn alloy electrodes after heat treatment at 600 °C, and with the etching by 10% HCl, nano layered structure was formed on the surface of the porous skeleton. Compared with the nickel foam, the surface area of the NiZn foam alloy electrode became larger, and the nano pore structure had good catalytic activity. At current density of 200 mA·dm^−2^, the hydrogen evolution overpotential of the NiZn foam alloy electrodes were reduced by 222 mV and 276 mV, respectively, through heat treatment of 600 °C and etching in 10% HCl solution, which indicated that the hydrogen evolution overpotential was effectively reduced because of the composite nano porous structure, while the activity of hydrogen evolution of the electrodes was obviously improved.

## Introduction

Hydrogen is a good clean fuel with a combustion value of up to 142.35 kJ·kg^−1^ and the combustion product is only the water without causing any environmental problems^1,2^. Electrolysis of water is an important way to obtain hydrogen on a large scale. The performance and price of the electrode material are the keys to achieve the industrialization for hydrogen production by electrolysis. The metal alloys are usually used as the electrode materials which have different characteristics of electronics and structures from the pure metals. In addition, increasing the exposed surface of the electrode materials can also improve the power efficiency^3–5^. Therefore, nickel, iron, platinum and other metals have been studied for the production of hydrogen evolution electrode^6,7^.

Foam nickel has lower mechanical and corrosion resistance with respect to bulk Ni. However, beacause of its large specific surface area, more and more persons studied it^8–10^. The catalyst carrier with foam nickel as the electrode of the electrolytic cell can effectively reduce the hydrogen evolution potential, and the energy conversion efficiency can be further improved by depositing nanomaterials on the foam nickel^11–15^. Ouyang *et al*.^11^ deposited a Ni_3_S_2_ nano-rod array on the surface of the foamed nickel by one-step hydrothermal method. Under 10 mA·cm^−2^ in 1 M KOH solution, the hydrogen evolution potential and oxygen evolution potential of the electrode surface were 200 mV and 217 mV, respectively. Tang *et al*.^12^ deposited a Ni_3_S_2_ nano-sheet array on a foamed nickel by hydrothermal method. In alkaline media, the overpotentials of the formed material at 10 mA·cm^−2^ and 100 mA·cm^−2^ were 123 mV and 260 mV, respectively. Although the results of the preparation and hydrogen evolution of foam nickel-based materials have been achieved^16–24^, it is still a challenge on how to reduce the over-potential of electrochemical hydrogen production on nickel electrodes.

Zn is a typical amphoteric metal that is chemically active in the alloy materials. So, the electrodeposition of Zn and Ni has generated a great amount of interest. The coatings of nickel electrodes with Zn prepared on some metal substrates were studied^25–32^. However, the NiZn foam alloy electrodes on the foam nickel substrates have not been studied extensively. In this paper, composite porous foam NiZn alloy electrode with nanoporous structures was prepared by the combination of electrodeposition, heat treatment and etching in HCl solution. The specific surface area of the electrode is increased. At the same time, the overpotential of hydrogen evolution of the prepared electrode is reduced.

## Experimental

### Preparation of electrodes

**Figure 1:**
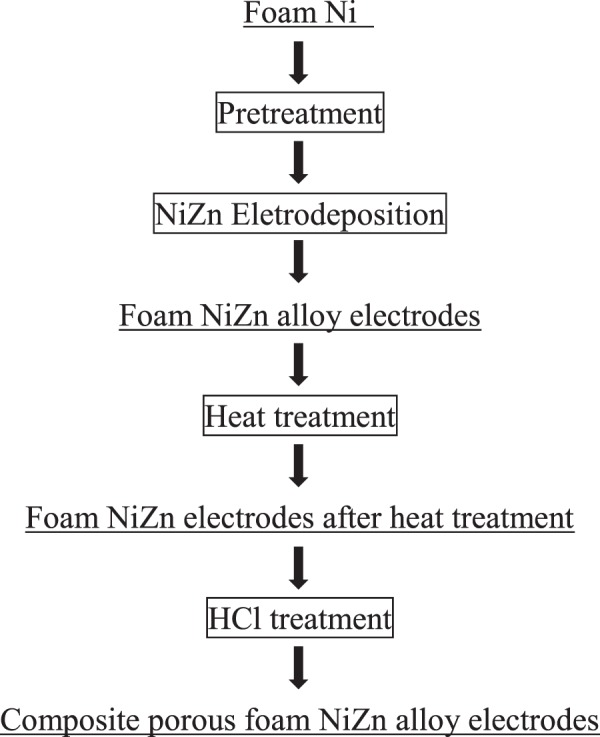
Process chart of preparing composite porous foam NiZn alloy electrodes.

The scheme of the preparation of porous foam NiZn alloy electrode was shown in Fig. 1. The process was described as follows.

1. Pretreatment of foam nickel: Foam nickel with thickness of 2 mm was used as the substrate, cleaned by acetone, soaked and degreased in alkaline solution (60 g·L^−1^ NaOH, 15 g·L^−1^ NaPO_4_, 30 g·L^−1^ Na_2_CO_3_ and 5 g·L^−1^ Na_2_SiO_3_) and then soaked in 10% dilute hydrochloric acid to remove the surface oxide.
2. The nickel plate was used as the anode, and the foam nickel substrate was used as the cathode. The electrodeposition was carried out with the process parameters listed in Table 1 to obtain the foam NiZn alloy electrodes.

**Table 1:** Process parameters of foam NiZn alloy electrodes.

Chemical reagents	Parameters
ZnCl_2_	40 g·L^−1^
NiCl_2_·6H_2_O	60 g·L^−1^
NiSO_4_·6H_2_O	180 g·L^−1^
NH_4_Cl	40 g·L^−1^
H_3_BO_3_	30 g·L^−1^
pH	5–6
Cathode-current density	40 mA·cm^−2^
Time	40 min
Temperature	30 °C
3. The foam NiZn alloy electrode was heat treated in a hydrogen atmosphere at a constant temperature of 500 °C, 600 °C and 700 °C, respectively, for 2 hours to obtain a higher mechanical strength and a better antioxidant performance.
4. The foam NiZn alloy electrode was etched in 10% HCl for 2 hours to remove part of the Zn.

### Test Methods

The surface morphology and energy spectrum of the electrode were observed by scanning electron microscopy (SEM, Nova Nano SEM 430). The specific surface area and pore size of the electrode were measured with Kubo-X 1000 multi-station microporous surface area analyser. The composition of the alloy was analyzed by X Pert - MRD diffractometer. The steady-state cathodic polarization curve of the electrode in a 30 wt% KOH solution at a temperature of 30 °C was obtained on a CHI660D electrochemical workstation with a scanning speed of 1 mV·s^−1^. The work station used a three-electrode system. A Pt electrode of large area was used as the auxiliary electrode. Saturated calomel electrode was used as the reference electrode. Three kinds of electrodes, i.e., a foam Ni electrode with a size of 10 mm × 10 mm, a foam NiZn alloy electrode after heat treatment and a foam NiZn alloy electrode treated with HCl, were tested as the working electrodes.

## Results and Discussion

### Morphology and energy spectrum analysis

**Figure 2:**
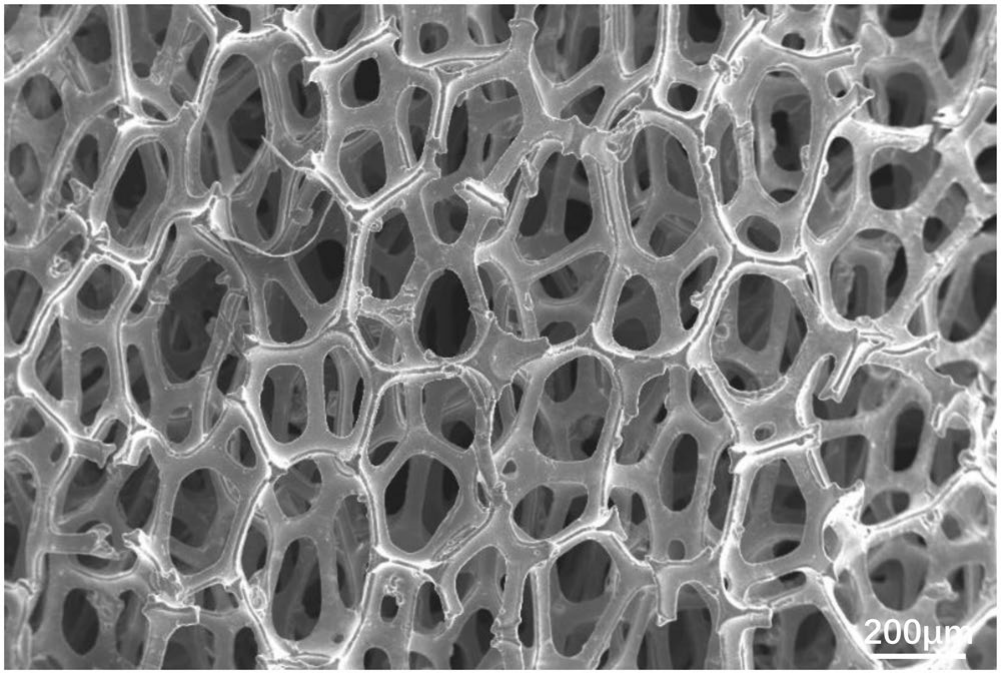
SEM image of foam Ni.

Figure 2 shows the SEM morphology of the foam nickel substrate. The pore sizes of the mesh structure are in the range of 100–800 μm.

**Figure 3:**
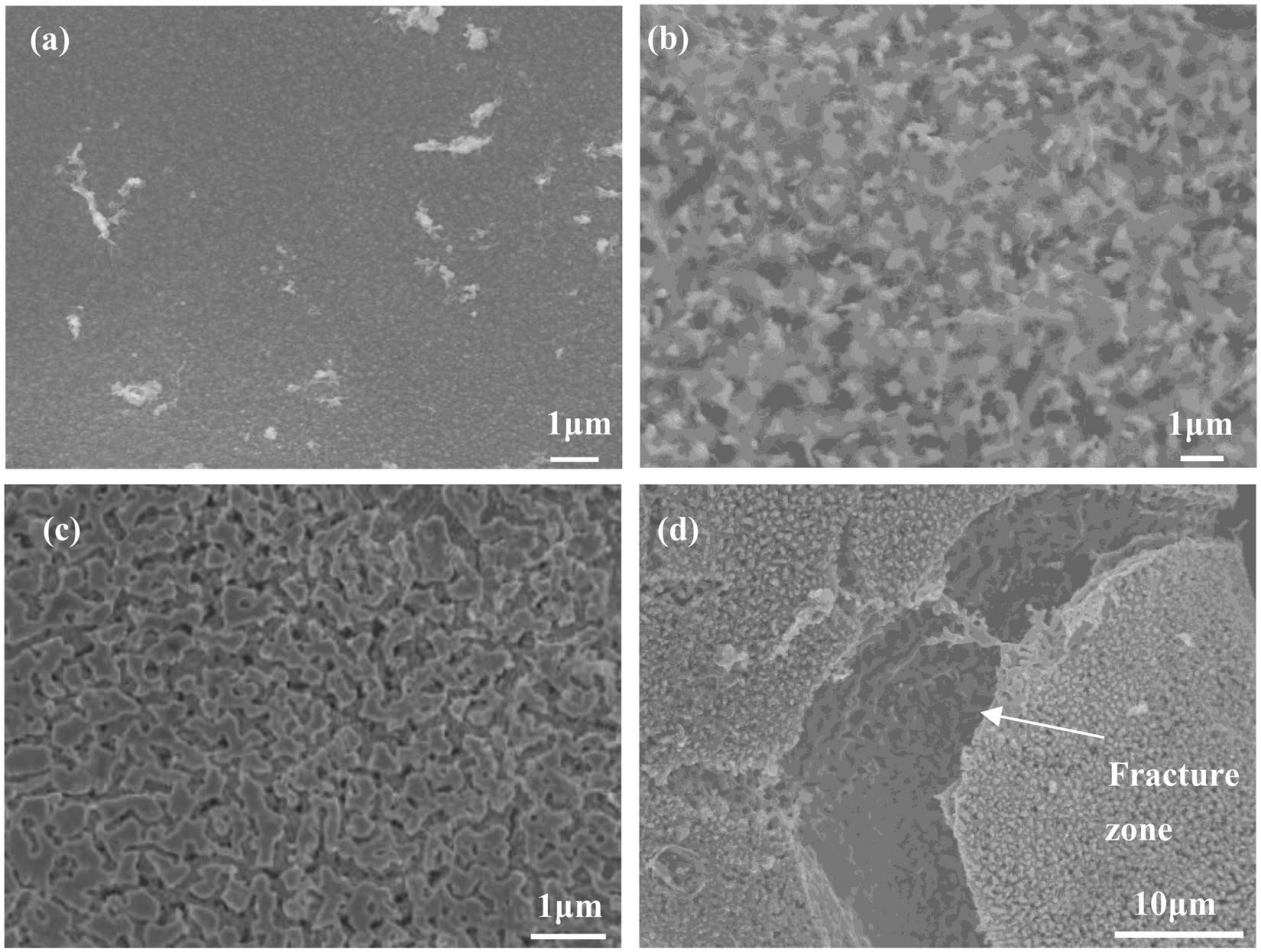
SEM images of foam NiZn alloy electrodes treated by different temperatures: (**a**) untreated, (**b**) 500 °C, (**c**) 600 °C, (**d**) 700 °C.

Figure 3 shows effect of temperature after the heat treatment. The surface of the foam NiZn alloy electrode after electrodeposition is uniform, as shown in Fig. 3a. However, after heat treatment at 500 °C, the electrode surface has pores, as shown in Fig. 3b. This is mainly due to the fact that the Zn layer is diffused into the pure Ni layer of the foam nickel substrate to form NiZn alloy, while the Ni layer remains solid in the alloy coating, and does not enter the foam nickel substrate layer with Zn, resulting in the occurrence of pores. With the heat treatment at 600 °C, the melting rate of Zn is increased and thus its diffusion to the Ni layer is accelerated. The pore structure is more obvious and the pore depth is increased, as shown in Fig. 3c. Figure 3d shows that the fracture phenomenon of the NiZn alloy coating occurs, when the temperature reaches 700 °C.

The EDS energy datas of foam NiZn alloy electrodes treated at different temperatures are shown in Table 2. Without heat treatment, the Zn content on the alloy surface is 71%. After heat treatment, the surface of the alloy layer is mainly rich in Ni, and the Zn content decreases with the increase of the heat treatment temperature. It is proved that Zn in the alloy layer melts and diffuses into the pure Ni layer, and the diffusion degree increases with the increase of the temperature.

**Table 2:** EDS datas of foam NiZn alloy electrodes treated at different temperatures.

Elements	Untreated/Wt%	500 °C/Wt%	600 °C/Wt%	700 °C/Wt%
Ni	29	28	69	88
Zn	71	72	31	12

**Figure 4:**
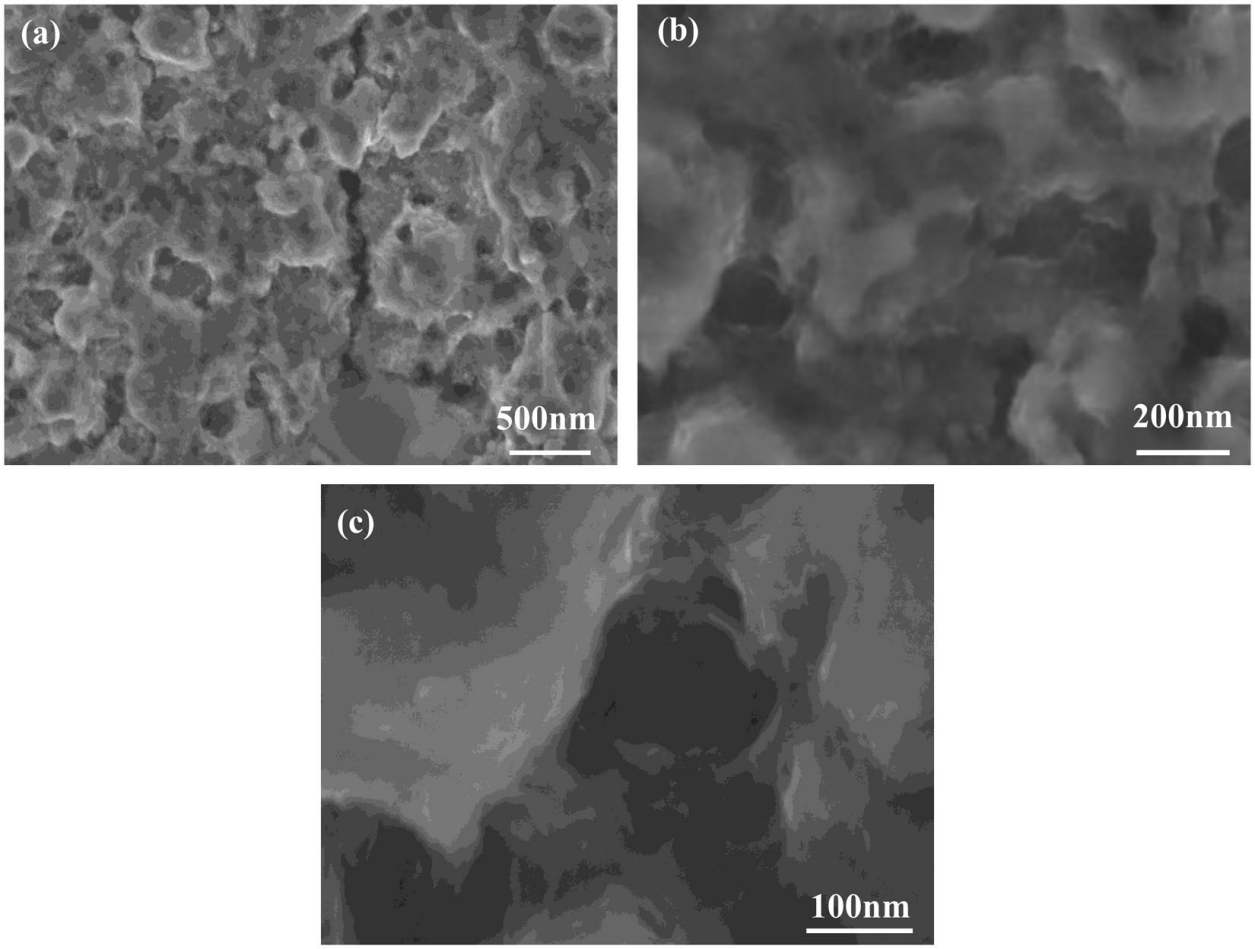
SEM images of foam NiZn alloy electrodes treated in HCl (**a**) 500 nm, (**b**)200 nm, (**c**) 100 nm.

Figure 4 shows the surface variation of the foam NiZn alloy electrode when it was etched in 10% HCl for 2 h. Most of the zinc in the foam NiZn alloy electrode was etched away, and a composite porous foam alloy electrode with nanoporous structure was formed on the surface of the coating. The nano-lamellar structure can further increase the specific surface area of the foam NiZn alloy electrode.

The data of EDS energy spectrum of the foam NiZn alloy treated in HCl with different time period are shown in Table 3. At the beginning of the treatment, the Zn content in the alloy coating is higher and the etching rate of Zn is faster. As the amount of Zn decreases, the etching rate slows down. After 60 min, the Zn content in the alloy layer is reduced to 12%. After 90 min, the Zn content in the alloy coating is almost constant. After 120 min, the Zn content in the NiZn electrode is 8% and the Ni content is 92%.

**Table 3:** EDS datas of foam NiZn alloy electrodes treated in HCl with different time period.

Elements	Foam NiZn + 600 °C	Foam NiZn + 30 min HCl	Foam NiZn + 60 min HCl	Foam NiZn + 90 min HCl	Foam NiZn + 120 min HCl
Ni	69	82	88	91	92
Zn	31	18	12	9	8

### Specific surface area and pore size

Table 4 shows the specific surface area and pore size of the foam nickel substrate and the foam NiZn alloy electrode after different treatments. The average pore size of the foam NiZn alloy electrode after heat treatment at 600 °C is 67 nm, and the specific surface area of that is significantly increased and nearly 29 times higher than that of the foam nickel substrate. After the treatment in HCl solution, the nano-porous structure is formed on the surface of the coating, which further reduces the average pore size of the electrode. And the surface area of the electrode is nearly 25 times higher than that of the foam NiZn alloy electrode after heat treatment.

**Table 4:** Specific surface area and pore size of the foam nickel substrate and the foam NiZn alloy electrodes after different treatments.

Electrodes	Foam Ni substrate	Foam NiZn + 600 °C	Foam NiZn + HCl
surface area**/**(m^2^·g^−1^)	0.059	1.70	42
Pore diameter**/**nm	—	67	34

### XRD analysis

**Figure 5:**
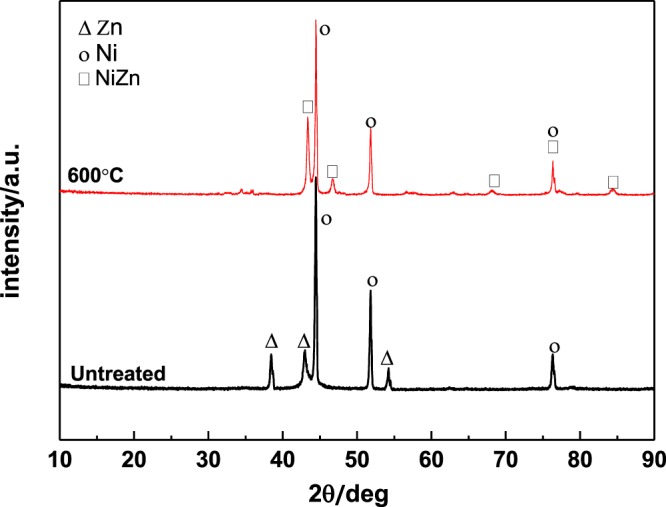
XRD patterns of the foam NiZn alloy electrodes before and after heat treatment at 600 °C.

Figure 5 shows the XRD pattern of the non-heat treated foam NiZn alloy electrodes and that after heat treatment at 600 °C. Before heat treatment. Ni and Zn peaks can be found in the XRD patterns of the electrode samples. After heat treatment, the three main peaks of Zn disappear, and the diffraction peaks of NiZn appear, which indicates that the NiZn alloy crystals are formed in the foam NiZn electrodes after heat treatment at 600 °C.

### Electrocatalytic characteristics for hydrogen evolution reaction

**Figure 6:**
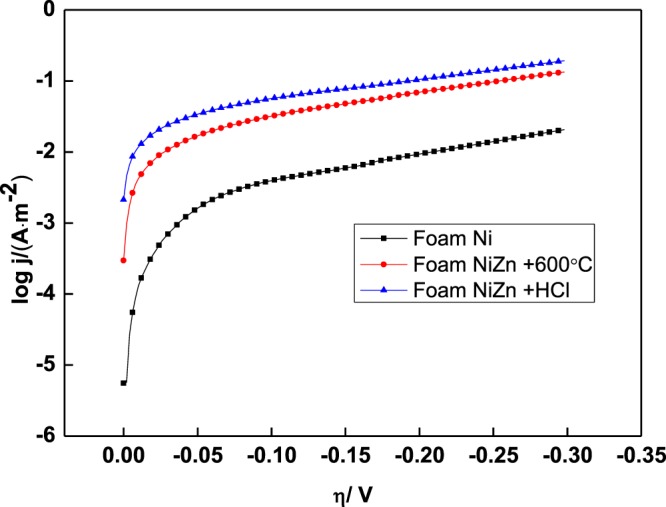
Cathodic polarization curves of the foam NiZn alloy electrodes with the different treatments.

Figure 6 shows the cathodic polarization curves of the foam NiZn alloy electrodes with the different treatments.

According to the Tafel equation

1 $$\eta =a+b\,\mathrm{log}\,j$$

with

2 $$a=-\,\frac{{\rm{2}}{\rm{.3RT}}}{\alpha {\rm{nF}}}\,\mathrm{log}\,{j}^{0}$$

and

3 $$b=\frac{{\rm{2}}{\rm{.3RT}}}{\alpha {\rm{nF}}}$$

In the formula above, *η* is the hydrogen evolution potential, *j* is the reaction current, a is the value related to the electrode material properties, the electrode surface state, the solution composition, the temperature, etc, *α* is the transfer coefficient, *j*^0^ is the exchange current density, and *b* is the tafel slope. In the cathodic polarization curve, a Tafel region with a good linearity is obtained, and its straight line is extended to intersect with a straight line of *η* = 0. The current density at this point is the exchange current density *j*^0^, which is the tafel slope *b*. Using *j*^0^ to characterize the catalytic activity of the electrode material, the greater the *j*^0^, the higher the catalytic activity of the electrode.

It can be seen from the Fig. 6 that, at the same current density, the polarization potential of foam NiZn alloy electrode with 600 °C treatment is significantly reduced compared with that of the foam Ni substrate. This shows that the higher hydrogen evolution activity is mainly caused by the heat treatment. The pores with an average pore size of 67 nm formed in the alloy coating, increase the specific surface area of the electrode and reduce the hydrogen evolution potential.

It can be seen from Table 5 that, the hydrogen evolution potentials of the two foam NiZn alloy electrodes after 600 °C treatment and HCl treatment are reduced by 222 mV and 276 mV at 200 mA·dm^−2^, respectively, compared with the foam nickel electrode. It indicates that the composite porous foam alloy electrode with nano-pore structure can significantly reduce the hydrogen evolution potential and improve the hydrogen evolution activity of the electrode.

**Table 5:** Overpotentials at 200 mA·dm^−2^ and exchange current density of the obtained electrodes.

Electrodes	Foam Ni substrate	Foam NiZn + 600 °C	Foam NiZn + HCl
η_200_/mV	582	360	306
**j**_**0**_**/**(A·cm^−2^)	9.2 × 10^−4^	10.1 × 10^−2^	2.3 × 10^−2^

The second line in Table 5 shows the exchange current density *j*^0^ of the electrode based on the polarization curve. As can be seen from it, both of the 600 °C treatment and the HCl treatment can increase the exchange current density of the foam nickel electrode. The exchange current density of the foam NiZn alloy electrode is 2.3 × 10^−2^ A·cm^−2^, which is much larger than that of foam Ni. The NiZn alloy electrode has higher hydrogen evolution activity after the HCl treatment.

The charge transfer control process and the diffusion process are two control steps in the hydrogen evolution process of the nickel electrode, the former being, the rate control step. The foam NiZn alloy electrode treated in HCl is a composite porous foam with nanoporous structure. Its good electrocatalytic ability is derived from the faster charge transfer rate during the hydrogen evolution reaction process, and more electrochemically active sites provide more reactive centers to increase the charge transfer rate. With the increase of the electrode surface porosity, the specific surface area increases, and its electrocatalytic activity increases under alkaline condition. Increasing the specific surface area of the electrode not only reduces the true current density in the electrolysis, but also reduces the hydrogen evolution potential of the electrode during the electrolysis reaction, and provides more hydrogenation activity centers, thus improving the electrocatalytic efficiency of hydrogen evolution. In addition, nickel has been widely used in the water electrolysis industry because there are unpaired electronson the 3d track of the outer layer of nickel atom as the transition metal, which makes it easy to form Ni-H adsorption bonds with hydrogen atom with the 1 s orbital electrons. However, to obtain highly active hydrogen evolution electrode, the good desorption capacity is also needed, which requires the use of alloying way to change the state of outer electrons of nickel atoms. Therefore, Ni-Zn alloying can reduce the nickel atoms on the surface of the rich d electrons and the active hydrogen atoms between the bonding capacity, and improve the ability of active hydrogen desorption.

## Conclusions

According to the analyses above, it is concluded that:

1. The NiZn alloy electrode with pore structure on the surface of the electrode was prepared by the method of electrodeposition and heat treatment. The optimum heat treatment temperature is 600 °C.
2. On the basis of heat treatment, the nanoporous structure is formed on the surface of alloy pore skeleton through etching in 10% HCl, resulting in a kind of composite porous foam alloy material, which further increases the specific surface area of the foam NiZn alloy electrode.
3. At the current density of 200 mA·dm^−2^ the hydrogen evolution potential of the foam NiZn alloy electrode after HCl treatment is 276 mV, which is lower than that of the foam nickel electrode, and the exchange current density is 2.3 × 10^−2^ A·cm^−2^, It is shown that the composite porous foam alloy electrode with nano-pore structure can improve the hydrogen evolution activity of the electrode.
